# Investigating Alternative Organic Ingredients for Sustainable Meagre (*Argyrosomus regius*) Aquaculture: Effects on Growth, Gut Microbiota and Plasma Biochemistry

**DOI:** 10.1155/anu/6697713

**Published:** 2025-11-10

**Authors:** Elisa Benini, Arianna Marchi, Francesco Dondi, Maria Giulia Ferrari, Phelly Vasilaki, Daniel Scicchitano, Giorgia Palladino, Marco Candela, Pier Paolo Gatta, Alessio Bonaldo, Luca Parma

**Affiliations:** ^1^Department of Veterinary Medical Sciences, University of Bologna, Ozzano dell 'Emilia, Italy; ^2^Irida S.A., Nea Artaki Evia, Greece; ^3^Department of Pharmacy and Biotechnology, University of Bologna, Bologna, Italy; ^4^Fano Marine Center, Fano, Italy

**Keywords:** aquafeed, organic aquaculture, organic pea protein, organic seaweed, organic yeast

## Abstract

The increasing global demand for sustainable seafood calls for innovative solutions that reduce reliance on marine resources. Organic aquaculture, with its focus on environmental sustainability and animal welfare, represents a promising avenue. However, one of the major constraints to its expansion is the limited availability of certified organic feed ingredients. In this study, we evaluated the feasibility of replacing fishmeal (FM) in the diets of meagre (*Argyrosomus regius*), a promising aquaculture species, by increasing the inclusion of organic pea protein meal (0%, 7%, 18% and 27.5%), while supplementing all pea-protein enriched diets with fixed levels of functional organic ingredients (5% yeast and 2% brown seaweed). Four isoenergetic diets—C (control), PEA 7%, PEA 18% and PEA 27%—were administered to triplicate groups of 40 juvenile meagre over 85 days. We assessed growth performance, feed utilisation, body composition, plasma biochemistry, and gut microbiota composition. All pea protein inclusion levels (7%–27.5%) showed comparable growth to the control diet, with no significant differences in final body weight (PEA 7%: 173 ± 22.5 g; PEA 18%: 189 ± 11.7 g; PEA 27%: 178 ± 23.7 g vs. control: 171 ± 10.0 g) or feed conversion ratio (FCR), demonstrating successful FM replacement up to 27.5%. The PEA 18% group showed superior lipid utilisation with 109% ± 7.21% gross lipid efficiency (GLE; vs. 75.4% ± 10.8% in PEA 7% and 85.3% ± 5.47% in control) and optimal protein metabolism (albumin [ALB]: 0.71 ± 0.07 g/dL vs. control: 0.62 ± 0.05), indicating enhanced nutrient utilisation at intermediate inclusion. High pea inclusion (27.5%) significantly increased beneficial *Lactobacillus* (8.74% ± 3.30% vs. control: 4.92% ± 3.96%) while maintaining microbial diversity, suggesting pre-biotic effects without dysbiosis. Overall, the results demonstrated that organic pea protein as well as organic seaweed and yeast can be successfully incorporated into meagre diets without compromising growth, feed efficiency, or fish health. These findings highlight the potential of organic pea meal as a sustainable protein alternative to FM in organic aquafeeds for meagre.

## 1. Introduction

Overcoming the challenges of the FOOD 2030 strategy requires innovative solutions. This is crucial given the projected 60% increase in global food demand by 2050 [1–3]. These solutions must focus on creating sustainable, resilient and inclusive food systems that can meet the nutritional needs of a growing population while also protecting the environment and promoting economic development. However, while aquaculture is known to be an industry able to offer a sustainable solution to meet the growing demand for seafood, it often faces negative public perception [4–6]. Concerns range from environmental impacts, such as water pollution and habitat destruction, to animal welfare issues, including overcrowding and disease outbreaks [7–11]. Additionally, some consumers express concerns about the quality and safety of farmed fish, particularly regarding the use of antibiotics and artificial feed [12–15]. These perceptions can be influenced by sensationalised media reports and a lack of transparency within the industry [16]. Therefore, to ensure the sustainable growth of the aquaculture sector, it is imperative to proactively address these emerging challenges.

In this context, organic aquaculture emerges as a promising alternative, prioritising environmental sustainability by minimising pollution and chemical use, promoting biodiversity conservation through natural habitats and avoiding GMOs and ensuring animal welfare through humane practices [17–19]. The sector is rapidly expanding in Europe, driven by a growing consumer demand for sustainable and high-quality seafood [20, 21]. In the Mediterranean regions, organic aquaculture is focused on commercial species such as European sea bass and gilthead sea bream, especially Greece [22] and Italy [23]. In North Europe, Atlantic salmon [24] and rainbow trout [25] were well involved in organic aquaculture with good results on growth and health. While organic aquaculture offers numerous benefits, challenges persist, including higher production costs, limited technological innovation and low consumer awareness [26, 27]. To unlock its full potential, continued investment in research, consumer education, supportive policies and industry-academia collaborations are essential. By addressing these challenges, organic aquaculture can contribute significantly to achieving sustainable and resilient food systems in Europe.

However, one of the greatest challenges facing organic aquaculture is the limited availability of certified organic feed ingredients, which are essential to comply with strict organic standards [21]. These standards mandate that feeds be composed of organically certified materials, minimise the use of synthetic additives and processing aids, avoid misleading or non-natural substances and utilise gentle and non-invasive processing methods [28]. Regulation (EU) 2018/848 sets specific criteria for different fish species; for carnivorous species such as meagre, organic feeds may include certified organic fishmeal (FM) and fish oil (FO), as well as organic plant-based ingredients—though the latter are restricted to a maximum of 60% of the diet.

Within this framework, identifying versatile and sustainable protein sources is essential. Organic pea protein is especially promising due to its high digestibility, favourable amino acid profile and low levels of anti-nutritional factors [29]. Moreover, organic seaweeds, such as *Ascophyllum nodosum*, offer valuable functional properties, including pre-biotic effects, immune modulation and antioxidant activity, while also contributing key micronutrients like iodine and polysaccharides [30, 31]. In parallel, single-cell protein (SCP) from organic yeast has emerged as a novel and environmentally efficient ingredient with high protein content and additional benefits such as improving gut health, modulating the immune response and supporting nutrient absorption [32–37]. These ingredients, when used in synergy, can support the nutritional, functional and regulatory needs of organic aquaculture.

In addition to meeting compositional standards, ingredient selection must consider economic viability and environmental sustainability. Feed production represents a major share of aquaculture's environmental impact; for instance, in Atlantic salmon farming, feed contributes approximately 72% of the global warming potential, acidification and 100% of biotic resource use per tonne of harvested fish [38]. As such, the strategic inclusion of low-impact ingredients like organic plant proteins, seaweed and SCP is critical for reducing the ecological footprint of aquaculture and enhancing the long-term viability of organic systems.

Emerging species are another tool to make the aquaculture sector more sustainable, promoting the conservation of biodiversity with all benefits linked with a healthy environment [39]. Diversification in aquaculture plays a crucial role in enhancing the sector's sustainability and resilience [40, 41]. By cultivating a variety of species, aquaculture systems can mitigate risks associated with market fluctuations, disease outbreaks and environmental changes. Additionally, diversifying species can optimise resource utilisation, improve water quality and promote ecosystem health [42, 43]. Ultimately, a diversified aquaculture sector contributes to food security, economic growth and environmental conservation. Among the species targeted to diversify the aquaculture landscape, meagre (*Argyrosomus regius*), a carnivorous species native to the Mediterranean and Black Seas and the Atlantic coast of Europe, is emerging as a promising candidate for aquaculture diversification in the Mediterranean region [44]. Its rapid growth rate, suitability for aquaculture and high-quality flesh make it an attractive choice for producers and consumers alike [44]. While the aquaculture of meagre is still relatively young, it has shown significant potential for sustainable production. As research and technology continue to advance, meagre aquaculture is considered to play a significant role in meeting the growing global demand for seafood while reducing pressure on wild fish stocks [44]. Numerous studies have focused on the nutritional requirements and growth optimisation of meagre. In particular, protein requirements have been estimated to be around 40%–50%, with plant proteins capable of replacing up to 76.2% of fish meal without compromising growth [45]. Lipid requirements have been established at approximately 17%, with essential fatty acids, particularly *n*−3 LC-PUFAs, required at a level of 2.1% of dry weight [46].

In this context, the present study aimed to evaluate the effect of organic pea protein, brown seaweed and yeast as alternative ingredients to FM and FO in the diet of meagre juveniles, assessing their impact on growth performance, health and compliance with organic aquaculture standards.

## 2. Materials and Methods

### 2.1. Ethics Approval

All experimental procedures were evaluated by the Ethical-Scientific Committee for Animal Experimentation of the University of Bologna in accordance with European directive 2010/63/UE on the protection of animals used for scientific purposes (ID 113/2020-PR).

### 2.2. Experimental Diets

Four experimental diets, isoproteic and isolipidic (crude protein 46% and crude lipid 16%), were formulated and produced via industrial extrusion with a pellet diameter of 3 mm by Irida S.A. (Greece). The control diet (C) was included 58.5% of fish meal from trimmings and 9.3% of FO from trimmings, as well as organic wheat and soybean. The three experimental diets (named PEA 7%, PEA 18% and PEA 27%) contained increasing levels of organic pea protein, respectively, the 7%, 18% and 27.5%, in replacement of fish meal trimming. Additionally, all the three experimental diets include 5% of organic yeast, as well as 2% percent of brown seaweed *Ascophyllum nodosum* (Sealac, Ireland) as a health promoting ingredient [30–32]. Ingredients and proximate composition of experimental diets are shown in Table 1.

### 2.3. Fish and Rearing Trial

The experiment was carried out at the Laboratory of Aquaculture, Department of Veterinary Medical Sciences of the University of Bologna (Cesenatico, Italy). Meagre (*Argyrosomus regius*) specimens were obtained from Cromaris (Zadar, Croatia) and adapted to the laboratory facilities for 3 weeks. Thereafter, 40 fish (initial average weight: 33.25 ± 0.14 g) per tank were randomly distributed into 12 800 L square tanks with a conical base. Each diet was administered to triplicate groups randomly assigned, over a period of 85 days.

Natural seawater, maintained at 24 ± 0.5°C and a salinity of 25 ± 5.0 g/L, was used to rear meagre juveniles the entire trial. Each tank operated within a closed recirculating aquaculture system (RAS). The RAS system consisted of a mechanical sand filter (PTK 1200, Astralpool, Barcelona, Spain), ultraviolet lights (UV PE 45; Sita Srl, Barcelona, Spain) and a biofilter (PTK 1200, Astralpool, Barcelona, Spain) as described by Parma et al. [47]. Oxygen level was maintained at 8.0 ± 1.0 mg/L through a liquid oxygen system connected to a software controller (B&G Sinergia snc, Chioggia, Italy). Photoperiod was held constant at 12 h:12 h light:dark regime. Ammonia (total ammonia nitrogen ≤0.1 mg/L) and nitrite (≤0.2 mg/L) were spectrophotometrically monitored once a day (Spectroquant Nova 60, Merck, Lab business, Darmstadt, Germany), and sodium bicarbonate was added daily to keep pH at 7.8–8.0. Feed was provided to satiation by oversupplying the feed via automatic feeders by approximately 10% of the daily ingested ration, twice a day (8:30 and 16:30) for 6 days a week. Each meal lasted 1 h and the uneaten pellets of each tank were gathered, dried overnight at 105°C and their weight was deducted for overall calculation [48].

### 2.4. Sampling

At the beginning and the end of the experiment, all the fish in each tank were anaesthetised or euthanised with tricaine methanesulfonate (MS-222) at 100 or 300 mg/L and individually weighed [49]. Specific growth rate (SGR), feed intake (FI) and feed conversion ratio (FCR) were calculated. Proximate composition was determined at the beginning of the trial on a pooled sample of 10 fish and on a pooled sample of five fish per tank at the end of the trial. Protein efficiency ratio (PER), gross protein efficiency (GPE), lipid efficiency ratio (LER) and gross lipid efficiency (GLE) were calculated.

### 2.5. Performance Parameter Calculations

Growth performance parameters (SGR, FI and FCR), nutritional indices (PER, GPE, LER and GLE) and somatic indices (hepatosomatic index [HIS] and viscerasomatic index [VSI]) were calculated based on the formulae detailed in Parma et al. [50].

### 2.6. Proximate Composition Analysis

Diets and whole body of sampled fish were analysed for proximate composition. Moisture content was obtained by weight loss after drying samples in a stove at 105°C until a constant weight was achieved. Crude protein was determined as total nitrogen (N) by using the Kjeldahl method and multiplying the resulting amount of N by 6.25. Total lipids were determined according to Bligh and Dyer's (1959) extraction method [51]. Ash content was estimated by incineration to a constant weight in a muffle oven at 450°C [52].

### 2.7. Plasma Biochemistry

Plasma biochemistry was assessed from aliquot of 500 μL collected at the beginning and at the end of the trials. Levels of glucose (GLU), total protein (Total Prot), albumin (ALB), urea, creatinine, uric acid, total bilirubin (Tot Bil), aspartate aminotransferase (AST), alanine transaminase (ALT), alkaline phosphatase (ALP), creatine kinase (CK), lactate dehydrogenase (LDH), cholesterol, HDL, triglycerides, calcium (Ca^+2^), phosphorus (P), magnesium (Mg^+^), iron (Fe), sodium (Na^+^), potassium (K^+^) and chloride (Cl^−^) were detected using samples of 500 μL on an automated analyser (AU 400; Beckman Coulter) according to the manufacturer's instructions. Moreover, ALB/globulin (Alb/Glo ratio) and Ca^+2^/phosphorus ratio (Ca/P ratio) were calculated.

### 2.8. Gut Microbiota Analysis

Following the 85-day feeding trial, total DNA was extracted from 300 mg samples of individual distal gut content obtained from 60 fish (15 per treatment), as described previously by Parma [53]. To ensure characterisation of the autochthonous microbiota, all animals were fasted for 24 h prior to sampling. The DNA was quantified using a NanoDrop spectrophotometer (NanoDrop Technologies, Wilmington, DEU) and stored at −20°C. Next, the V3–V4 hypervariable regions of the 16S rRNA gene targeting bacteria were amplified using established primers and protocols [54] with small modifications. In particular, primer solutions were prepared at 2 µM by diluting 50 µM stocks into PCR grade water with 10% *w*/*v* solution of BSA (bovine ALB serum) to prevent possible inhibitors from interacting with DNA polymerase [54]. After amplification, purification, and library preparation following Illumina's guidelines, libraries were normalised, pooled, and sequenced on an Illumina MiSeq platform using a paired-end 2 × 250 bp protocol. Raw sequence data was processed using a combined PANDAseq and QIIME2 pipeline [55] (https://qiime2.org). High-quality reads were obtained by filtering for length (350–550 bp) and quality (max 3% error rate) using USEARCH [56]. DADA2 was used to clean and cluster the filtered reads into amplicon sequence variants (ASVs). Taxonomic assignment employed a hybrid VSEARCH and q2-classifier method trained on the SILVA 138.1 database [57]. To evaluate the diversity of bacterial communities within each sample (alpha diversity), three metrics were employed: Faith's phylogenetic diversity, Shannon entropy index, and the number of observed ASVs. Unweighted UniFrac distances were calculated to assess diversity between samples (beta diversity) and used for principal coordinates analysis (PCoA).

### 2.9. Statistical Analysis

All data are presented as mean ± standard deviation (SD). Tank was used as an experimental unit to evaluate growth performance. Furthermore, a pool of 10 fish was considered the experimental unit for the analysis of proximate composition and nutritional indices. Five individual fish were used for analysing somatic indices, blood biochemistry and gut microbiota community profiles in each experimental unit. The homogeneity of variance assumptions was validated for all data preceding ANOVA. Tukey's post hoc test was performed. All statistical analyses were performed using GraphPad 8.0.1. The differences among treatments were considered significant at *p* ≤ 0.05. Microbiota analysis and respective plots were produced using R software (https://www.r-project.org/) with ‘vegan' (http://www.cran.r-project.org/package-vegan/), ‘Made4' [58] and ‘stats' packages (https://stat.ethz.ch/R-manual/R-devel/library/stats/html/00Index.html). Data separation was assessed by a permutation test with pseudo-*F* ratios (function “Adonis” in “vegan” package). When required, Wilcoxon and Kruskal–Wallis test were used to assess significant differences in alpha diversity and taxon relative abundance between groups. When necessary, *p*-values were corrected for multiple testing with Benjamini–Hochberg method, with a false discovery rate (FDR) ≤0.05 considered as statistically significant.

## 3. Results

### 3.1. Growth

Summary of the growth index results are presented in Table 2. No statistical difference was observed among treatments for all the growth index analysed (IBW, FBW, WG, SGR, FI and FCR) as well as for survival (%). Although the survival rate in the control group (81.7% ± 13.8%) appeared lower than in the PEA-fed groups, no mortality-related patterns or pathological signs were observed. All fish were subject to the same environmental and handling conditions, and the variability is likely due to random mortality unrelated to dietary treatment.

### 3.2. Proximate Composition

Body composition, nutritional indices and somatic indices measured in meagre's specimens are shown in Table 3. No statistical differences were observed in whole body composition among treatments. Considering the nutritional indices, GLE was significantly influenced by the dietary treatment where fish fed PEA 18% diet present a higher GLE compared to PEA 7% and PEA 27%. Focusing on the somatic indices, results show no significant difference between diet for CF and VSI (*p* > 0.05), while the hepatosomatic induce was significantly affected by the dietary treatments (*p*=0.0200), where fish fed PEA 7% had a higher HIS compared to PEA 18%.

### 3.3. Plasma Results

Results of plasma biochemistry are showed in Table 4. Statistical differences were evaluated in GLU, Total Prot, ALB, Alb/Glo, current Ca^+2^ and Na^+^ parameters. GLU was higher in animals fed with control diets and lower in PEA 27% diet. Total Prot was lower in control diet and PEA 27% compared to PEA 18% diet. ALB showed higher values in animal fed with PEA 18% diet than control diet. Alb/Glo values were higher in animals fed with PEA 27% diet than C and PEA 7% diets. Current Ca^+2^ was lower in PEA 18% and PEA 27% diets compared to PEA 7% and Na was higher in C and PEA 17%.

A significant decrease in GLU levels was observed as the pea level increased (*p*=0.0013). The lowest GLU level was seen in PEA 27%. While, no significant differences were observed across the treatments (*p*=0.8413) for lactate.

Total Prot levels increased significantly with PEA inclusion, peaking at PEA 18% before dropping slightly in PEA 27% (*p*=0.0090). Similar to Total Prot, ALB and Alb/Glo ratio increased significantly with organic pea protein inclusion, particularly in PEA 18% (*p*=0.0081) for ALB and in PEA 27% (*p*=0.0068) for Alb/Glo ratio.

No significant effects were observed on urea (*p*=0.0811) and on creatinine (*p*=0.0519), as well as on Tot Bil, AST and ALT (respectively, *p*=0.5819, 0.8578 and 0.8124).

Moreover, no significant changes in enzyme activities (ALP, CK and LDH) as for lipid profile biomarkers (cholesterol, HDL or triglycerides). In relation to the electrolyte's concentration in plasma, a significant decrease in Na^+^ levels was observed with PEA 27% (*p*=0.0010) and a significant decrease was seen at higher organic pea protein levels (*p*=0.0467). Furthermore, other minerals (Mg, Fe, K and Cl) did not show significant changes through treatments.

### 3.4. Gut Microbiota

**Figure 1:**
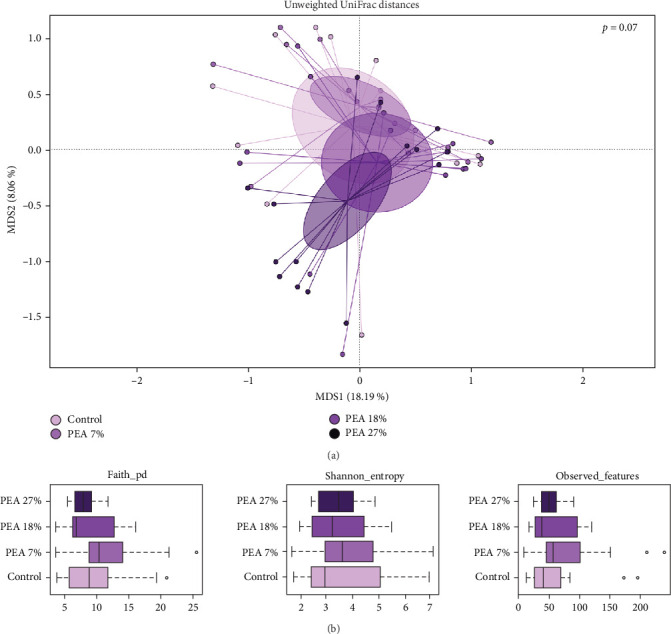
Beta diversity and alpha diversity of gut microbiota of meagre fed with experimental diets over 85 days. (A) Principal coordinates analysis (PCoA) based on unweighted UniFrac distances between gut microbiota composition of animals fed with experimental diets. No significant separations were highlighted (permutation test with pseudo-*F* ratios Adonis; *p*  > 0.05). (B) Alpha diversity boxplots showing the distribution of tree metrics assessed in the four experimental groups. No significant variations in all the metrics analysed were observed (*p*  > 0.05).

The 16S rRNA gene sequencing was performed on a total of 60 distal intestine content samples. Overall, both alpha and beta diversity of the gut microbiota was not influenced by dietary treatments, as shown in Figure 1.

**Figure 2:**
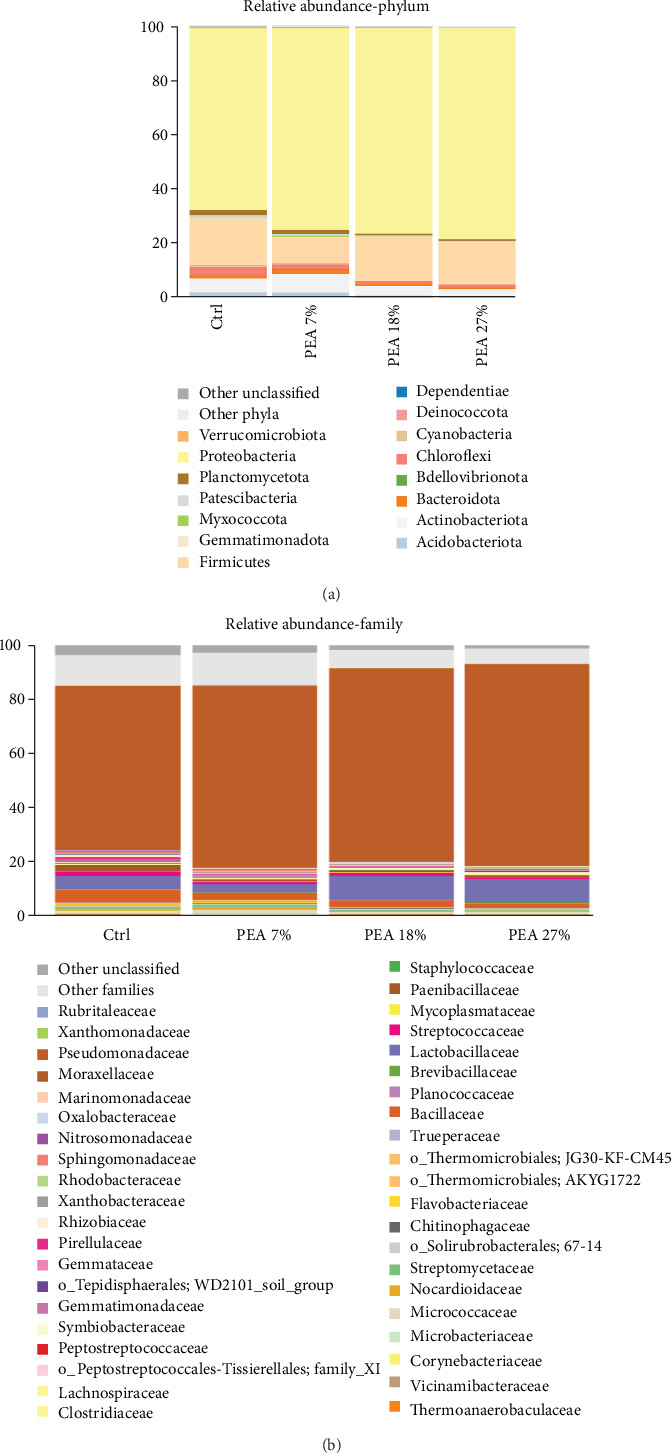
Microbiota composition of distal gut content of meagre fed with experimental diets. Bar plot summarising the microbiota composition at phylum (A) and family (B) level of fish intestinal content. Only phyla with a relative abundance ≥0.5% in at least 2 samples are shown, and families with a relative abundance ≥0.5% in at least 5 samples are shown.

Considering the overall gut microbiome analyses independently from the dietary treatment, when data have been analysed at phylum level (Figure 2A), the most abundant taxon observed was Proteobacteria (with an overall relative abundance mean of 74%), followed by Firmicutes (with an overall relative abundance mean of 15%).

Furthermore, the most represented families were *Pseudomonadaceae* (specific relative abundance: 73.7% ± 4.83% C; 60.9% ± 9.21% PEA 7%; 67.7% ± 6.72% PEA 18%; 71.5% ± 7.77% PEA 27%) and *Lactobacillaceae* (4.92 ± 3.96 C; 2.81% ± 0.94% PEA 7%; 8.83% ± 5.11% PEA 18%; 8.74% ± 3.30% PEA 27%), the latter belonging to Firmicutes phylum. The most abundant genus was *Pseudomonas* (with an overall relative abundance mean of 69%; Figure 2B).

**Figure 3:**
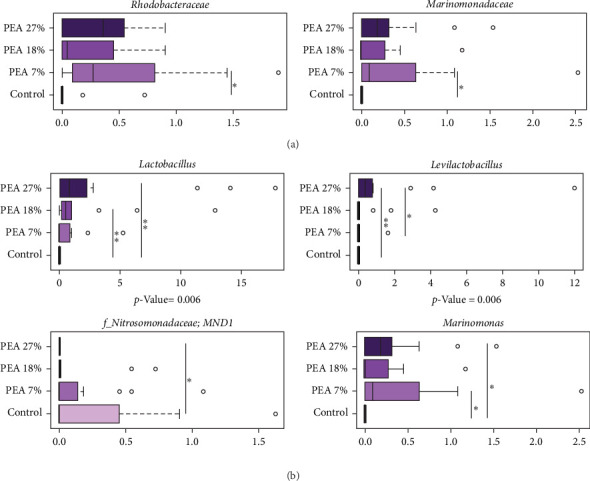
Taxonomic composition of bacterial communities in the distal gut content of meagre fed experimental diets. (A) The first two graphs (top) show family-level relative abundance boxplots, and (B) the remaining four graphs show genus-level relative abundance boxplots. Distributions of relative abundance of families and genera that showed significant variation between groups fed with different diets (Wilcoxon rank–sum test; *p* < 0.05). The central box of each box plot represents the interquartile range (25th–75th percentiles), and the black line inside each box indicates the median. Bold values indicate statistically significant differences between treatments (*p* < 0.05). *⁣*^*∗*^ indicates *p* < 0.05 (statistically significant difference), while *⁣*^*∗∗*^ indicates *p* < 0.01 (very significant).

Focusing on the specific variation between each group at family level (Figure 3A), we observed a significant increase in the relative abundance of *Rhodobacteraceae* and *Marinomonadaceae* genera in PEA 7% group compared to PEA 27% group (Wilcoxon rank–sum test, *p* < 0.05). At genera level (Figure 3B), the *Lactobacillus* and *Levilactobacillus* genera significantly increased in fish fed PEA 27% compared to the control diet (*p* < 0.01). Also, *Lactobacillus* resulted significantly increased in fish fed PEA 18% compared to the control diet (*p* < 0.01). On the other hand, we observed a significant decrease of *Nitrosomonadaceae*, in treated group PEA 27% compared to the Control group (*p* < 0.05), while *Marinomonas* increased in PEA 7% and PEA 27% groups compared to Control diet (*p* < 0.05; Figure 3B).

## 4. Discussions

Organic aquaculture is rapidly gaining momentum, driven by growing consumer demand for sustainable and environmentally friendly seafood products [59]. However, it faces several challenges, most notably in feed formulation. Regulatory restrictions, such as the prohibition of synthetic amino acids, significantly limit the ability to develop nutritionally balanced diets [21]. In addition, organic and protein-rich raw materials are both scarce and substantially more expensive than conventional alternatives. This limited availability continues to drive a strong reliance on FM and FO, representing a major obstacle to the development of cost-effective and truly sustainable organic feeds [60–62].

In this context, meagre emerges as a promising candidate for organic aquaculture due to its rapid growth, high-quality flesh and nutritional adaptability [44–46]. Moreover, the use of functional organic ingredients not only meets consumer expectations [59] but also improves fish resilience to environmental stressors, thereby supporting more sustainable farming practices [31, 63, 64]. In the present study, organic yeast (5%) and brown seaweed (*Ascophyllum nodosum*, 2%) were incorporated into all diets, with inclusion levels chosen based on prior research highlighting their nutritional and functional roles in aquafeeds [32, 65–67]. Specifically, dietary yeast has been shown to enhance immune function, promote beneficial gut microbiota and mitigate the adverse effects of plant-based ingredients in aquafeeds [32, 35–37, 48]. Similarly, *A. nodosum* is recognised for its pre-biotic and immunomodulatory properties in fish, even at low inclusion rates [66, 68]. Additionally, pea protein has gained attention as a promising plant-based protein source in aquafeeds. It is nutritionally balanced, compliant with organic regulations, environmentally sustainable, and biologically well-tolerated. Its use has been extensively studied not only in aquaculture species such as salmon [69] and crayfish [70], but also in terrestrial animals like dogs [71], pigs [72] and even humans [73].

Based on the results of this study, growth parameters and FI, key indicators of feed formulation efficiency, showed no significant statistical differences among the experimental groups with alternative dietary formulations. Similar findings were reported in a previous study on meagre fed with an organic selenium supplement [74] as well in a study on the effect of dietary green pea protein and seaweed in replacement of FM in gilthead seabream [75]. By comparing the values from both studies, the results presented in this article can be considered representative of standard performance benchmarks for meagre under these dietary conditions.

Furthermore, data on biometric parameters, body composition and nutritional index did not show any significant difference between group, with the only exception of the nutritional index GLE, which was higher in PEA 18% compared to PEA 7% and PEA 27%, and HSI, significantly higher in PEA 7%. These results showed differential lipid utilisation efficiency and liver response across dietary treatments. Higher GLE in the PEA 18% group likely reflects more effective lipid utilisation and metabolism. For instance, Gou et al. [76] demonstrated in the ciprinid *Onychostoma macrolepis* that moderately higher dietary lipids (~9%–11%) significantly boosted antioxidant responses and liver functionality by upregulating key lipid-processing enzymes such as lipoprotein lipase and carnitine palmitoyltransferase‑1, essential for fatty acid oxidation and tissue lipid deposition. Furthermore, that supplementing diets with 1 % lecithin in giant grouper (*Epinephelus lanceolatus*) significantly enhanced weight gain (~12%), elevated circulating triglycerides and improved lipid transport, highlighting lecithin's role in chylomicron formation and lipid mobilisation [77]. Together, these studies support our interpretation that intermediate inclusion of organic pea protein, when formulated with balanced lipid profiles and functional emulsifiers, can enhance lipid metabolism efficiency in meagre.

While, increased HSI in PEA 7% suggests potential liver stress or enhanced fat storage. The interplay between dietary lipid utilisation efficiency and liver function in fish; for instance, HSI, has been well-documented in aquaculture research [78–81]. Research on species like yellowtail kingfish (*Seriola lalandi*) [82, 83] and Nile tilapia [84, 85] shows that higher dietary lipid levels often lead to increased HSI due to enhanced lipid deposition in the liver. Specifically, yellowtail kingfish exhibited significant HSI changes linked to lipid content, suggesting liver stress or fat storage adaptations when lipid levels were elevated [86]. A similar trend was observed in tilapia, where higher lipid diets increased HSI and triglyceride levels in both plasma and liver [87]. Moreover, while higher lipid levels may improve feed efficiency (lower FCR), they can simultaneously elevate HSI, indicating a trade-off between growth optimisation and potential metabolic strain. These findings demonstrated the importance of balancing dietary lipids to support both growth performance and liver health. Nevertheless, although no statistically significant differences in growth performance or proximate composition were observed among the dietary treatments, the trends suggest potential benefits at the intermediate inclusion level (18% organic pea protein). The consistent growth across treatments supports the viability of incorporating plant-based ingredients without compromising fish performance or health. Thus, the lack of growth differences could be attributed to sufficient nutrient balancing in the diets, demonstrating that pea protein partially replaces FM effectively in this context.

Other indicator to detect animal health and how an experimental diet can influence it is plasma biochemistry. The observed changes in plasma GLU, protein markers and Na^+^ levels due to dietary treatments with different pea protein levels offer important insights into the physiological effects of plant-based protein inclusion. As pea meal contains a high concentration of carbohydrates, higher concentrations of GLU in the blood might have been expected due to the presence of specific anti-nutritional factors in peas, such as non-starch polysaccharides (NSPs), raffinose-family oligosaccharides (RFOs) and phytates, which are known to impair carbohydrate digestibility and nutrient absorption [88–90]. These compounds can increase intestinal viscosity, interfere with enzymatic hydrolysis and shift GLU fermentation toward the hindgut, collectively contributing to lower glycaemic responses. The significant reduction in plasma GLU observed in this study with increasing levels of organic pea protein may indicate a favourable modulation of carbohydrate metabolism [88]. This aligns with prior studies showing that pea-derived components, including protein hydrolysates, glycoproteins and dietary fibre, exert hypoglycaemic effects in both animal models and humans [91]. These effects are mediated through mechanisms such as inhibition of α-amylase and α-glucosidase, enhancement of insulin sensitivity and secretion, promotion of hepatic glycogen synthesis and regulation of GLU transporter expression (e.g., GLUT1) via the IRS–PI3K–Akt pathway [92]. Clinical trials have further shown that pea protein can reduce postprandial glycaemia and stimulate insulin production [93]. Therefore, the observed GLU-lowering effect in meagre may reflect adaptive metabolic responses to plant-based protein sources, supporting the functional role of organic pea protein in promoting energy homeostasis in aquaculture species.

Concurrently, the increase in Total Prot and ALB levels at the 18% organic pea protein inclusion points to enhanced protein metabolism and utilisation efficiency, which may be optimal at intermediate dietary levels. Likewise, the observed decline in plasma Na^+^ levels with PEA 27% inclusion may reflect minor effects on ion homeostasis, a phenomenon also reported in fish fed high plant-protein diets. Although still within normal physiological ranges, this suggests a potential interaction between diet and osmoregulatory mechanisms, meriting further exploration. Importantly, no adverse effects were detected in kidney or liver markers, suggesting that these shifts are adaptive rather than pathological. The replacement of FM with plant-based ingredients, such as organic pea protein, may influence ion homeostasis in fish [94]. This effect could result from differences in dietary mineral composition, reduced bioavailability of certain ions (e.g., Na^+^, K^+^ and Ca^+2^) and the presence of anti-nutritional factors that can interfere with intestinal ion absorption. Pea meal contains several well-known anti-nutritional factors that may interfere with carbohydrate digestion and metabolism in fish. These include NSPs, RFOs, phytates and trypsin inhibitors. NSPs and RFOs, in particular, increase intestinal viscosity and can reduce nutrient absorption by impairing the access of digestive enzymes to substrates [95]. Phytates may bind minerals and interfere indirectly with enzymatic activity and gut health, potentially altering carbohydrate and energy metabolism [88]. These compounds can shift the site of carbohydrate fermentation to the hindgut, producing volatile fatty acids instead of GLU, which contributes to reduced glycaemic response and explains the lower plasma GLU levels observed with increasing pea protein inclusion. Interestingly, carbohydrate metabolism is closely linked to lipid metabolism in fish through several physiological pathways [96, 97]. Reduced GLU availability due to impaired carbohydrate digestion may lead fish to rely more heavily on lipid oxidation for energy, thereby altering lipid utilisation patterns [98–100]. This shift could partially explain the enhanced GLE observed in fish fed the PEA 18% diet, as the body may compensate by mobilising and utilising lipids more efficiently. Thus, the observed changes in both GLU and lipid parameters may reflect a coordinated metabolic adjustment to the nutrient composition and digestibility of the diet. Additionally, dietary-induced changes in gut microbiota may modulate intestinal permeability and electrolyte transport mechanisms. Although plasma Na^+^ levels remained within physiological ranges, the significant reduction observed in fish fed higher levels of pea protein (PEA 27%) suggests a possible adaptive osmoregulatory response linked to dietary composition.

Although no significant differences were observed in overall gut microbial diversity, as confirmed by alpha and beta diversity metrics, the relative abundance of certain bacterial genera exhibited subtle but statistically significant shifts in response to dietary treatments. In particular, fish fed higher levels of organic pea protein (PEA 18% and PEA 27%) showed an increased abundance of *Lactobacillus* and *Levilactobacillus*, genera known for their beneficial roles in gut health and mucosal immunity. However, these findings should be interpreted with caution, as they occurred within the context of an otherwise stable microbial community structure. The absence of broad-scale microbial disruption or dysbiosis suggests that these taxonomic shifts likely reflect adaptive or transient microbial responses to dietary fibre and plant-based compounds, rather than major community restructuring. These results are consistent with previous studies reporting minor modulation of specific gut taxa without large changes in overall diversity in fish fed plant-based or functional diets. This adaptation has been documented for Atlantic salmon [101] as well as for other fish species such as rainbow trout [102], sablefish (*Anoplopoma fimbria*) [103], European seabass [104] and Gilthead seabream [75]. Overall, the data support the compatibility of organic pea protein and associated ingredients with gut microbial homeostasis in meagre.

Moreover, it is known that pea meal has a lower level of anti-nutritional factors compared to other legumes [105–107] enhance its potential as an aquafeed component, promoting sustainable aquaculture without compromising gut health. Furthermore, an increase in abundance of Lactic acid bacteria (LAB) bacteria (*Lactobacillus* and *Levilactobacillus*) in meagre fed PEA 27% indicate potential pre-biotic effects of higher pea protein inclusion. LAB are natural residents of the fish gut with the ability to adhere and colonise, providing multiple benefits. They support gastrointestinal development, enhance digestive function, maintain mucosal tolerance, strengthen immune responses and protect against bacterial pathogens, making them valuable for gut health [108]. Comparable effects of the pea protein have been observed in human's gut intestinal tract, where glycated pea proteins influenced the growth of beneficial gut bacteria like *Lactobacilli* and *Bifidobacteria*. This was accompanied by a shift in bacterial metabolites; notably, an increase in short-chain fatty acids (SCFAs) such as acetate, propionate, lactate and butyrate. Intestinal bacteria were found to metabolise glycated pea proteins, suggesting that the energy within these proteins, which is partially inaccessible to digestive enzymes, could be reclaimed through microbial fermentation, highlighting their functional role in gut health. Additionally, positive effects of the use of pea protein on the gut microbiome have been documented also in chicken as well as in rodents and pigs (reviewed in [109]). Considering the fish realm, dietary inclusion of pea protein in species like Atlantic salmon [110, 111] and rainbow trout [112] have shown effects on gut microbiota, including LAB populations, enhancing gut health and immune responses. Research indicates that diets incorporating 18% unprocessed pea meal [113] or 20% processed pea protein [114] do not induce dysbiosis in Atlantic salmon. This is significant because vegetable-derived proteins, particularly soymeal, are often associated with gut health issues like dysbiosis. This finding is particularly relevant for salmonids, where vegetable-derived proteins (especially soymeal) are well-documented to induce gut dysbiosis and enteritis [101, 114]. While Mediterranean species like gilthead seabream and European seabass generally show greater tolerance to plant-based ingredients [115, 116], the absence of dysbiosis with pea protein in meagre, a promising candidate for Mediterranean aquaculture, confirms its advantages over conventional alternatives. These findings underscore that the inclusion of pea protein, particularly at higher levels, not only enhances gut health by selectively promoting beneficial LAB, demonstrating its functional role as a pre-biotic across various species, including fish, humans and terrestrial animals, without inducing dysbiosis. Overall, the microbiota findings suggest that high organic pea protein inclusion in the diet may selectively encourage the growth of beneficial bacterial taxa.

Notably, despite their inclusion in small quantities, the organic diets in this study incorporated functional ingredients, 5% yeast and 2% brown seaweed (*Ascophyllum nodosum*), reflecting current industry practices for sustainable aquafeeds. While these inclusions prevented isolation of their individual effects due to the study design, their established functional properties in other systems are noteworthy. Yeast is recognised for its nutritional value [35, 117], environmental benefits [118, 119] and ability to counteract plant-based diet challenges in salmonids [36, 65] and modulate pathogenic bacteria in catfish [37]. Similarly, *A. nodosum* provides essential nutrients [30], immunomodulatory compounds and pre-biotic effects [31, 68]. In our trial, the maintained growth performance (FBW range: 171–189 g across diets) and gut microbiota stability (e.g., increased Lactobacillus at 27.5% pea inclusion: 8.74% ± 3.30% vs. control 4.92% ± 3.96%) suggest these ingredients may have synergistically supported nutrient utilisation, as evidenced by the 28% higher lipid efficiency in PEA18% (109% ± 7.21% vs. control 85.3% ± 5.47%). However, their specific contributions warrant future research with ingredient-excluded controls.

## 5. Conclusions

The results of this study support the use of organic pea protein, brown seaweed and yeast as effective alternatives to FM and FO in the diet of meagre juveniles, demonstrating their potential to sustain growth performance and health while remaining in compliance with organic aquaculture standards. Specifically, growth, FI and health parameters remained unaffected, underscoring the feasibility of using pea protein without compromising fish performance. Optimal organic pea protein inclusion levels, such as 18%, showed enhanced protein efficiency and lipid metabolism without inducing dysbiosis or liver stress. The microbiota findings further emphasise the pre-biotic effects of higher pea inclusion, selectively promoting beneficial LAB, which contribute to gut health and immune resilience. These outcomes align with similar benefits observed in other species, suggesting that organic pea protein in combination with organic yeast and brown seaweed are not only a sustainable alternative but also functional feed ingredients that can supports the environmental and economic goals of aquaculture. This study provides a foundation for advancing organic and sustainable feed formulations in aquaculture.

## Figures and Tables

**Table 1:** Ingredients and proximate composition of the experimental diets.

Composition (%)	C	PEA 7%	PEA 18%	PEA 27%
Ingredients (% of the diet)				
Fish meal trimmings	58.5	50.0	41.0	33.0
Fish oil trimmings	9.50	10	10.0	10.5
Organic soybean meal	15.5	15.5	15.5	15.5
Organic wheat	16.0	10.	8.00	6.00
Organic pea protein	—	7.00	18.0	27.5
Organic yeast	—	5.00	5.00	5.00
Seaweed *Ascophyllum nodosum*	—	2.00	2.00	2.00
Organic premix (vitamins–minerals)	0.60	0.60	0.60	0.60
Natural antioxidant	0.03	0.03	0.03	0.03
Proximate composition (% on a wet weight basis)				
Moisture	8.5	8.5	8.7	8.5
Protein	45.8	45.6	45.6	45.7
Lipid	15.8	15.7	15.9	15.9
Ash	11.6	13	11.2	10

*Note*: Formulated according to the EU Reg. 2018/848, suitable for organic fish farming. Nutritional requirements for this species have been respected in order to guarantee the health of animals.

**Table 2:** Growth performance and feed intake of meagre fed experimental diets over 85 days.

Growth parameters	C	PEA 7%	PEA 18%	PEA 27%	*p*-Value
IBW	33.7 ± 0.06	33.3 ± 0.20	33.2 ± 0.06	33.2 ± 0.15	0.4058
FBW	171 ± 10.0	173 ± 22.5	189 ± 11.7	178 ± 23.7	0.5360
WG	139 ± 9.99	140 ± 22.5	156 ± 11.7	145 ± 23.9	0.6713
SGR	1.93 ± 0.07	1.93 ± 0.15	2.04 ± 0.07	1.97 ± 0.17	0.6754
FI	1.82 ± 0.04	1.82 ± 0.16	1.76 ± 0.03	1.77 ± 0.06	0.7828
FCR	1.21 ± 0.09	1.17 ± 0.20	1.09 ± 0.06	1.13 ± 0.07	0.6399
Survival	81.7 ± 13.8	90.0 ± 15.2	93.3 ± 1.40	87.5 ± 5.00	0.5962

*Note:* Data are given as the mean ± SD (*n* = 3). SGR (%/day) = 100 × (ln FBW − ln IBW)/days. FI (g feed/fish) = g feed ingested/number of fish. FCR = feed intake/weight gain. Survival, survival (%).

Abbreviations: FBW, final body weight; FCR, feed conversion rate; FI, feed intake; IBW, initial body weight; SGR, specific growth rate; WG, weight gain (g).

**Table 3:** Body composition, nutritional indices and somatic indices measured in meagre.

Body composition	Parameters	C	PEA 7%	PEA 18%	PEA 27%	*p*-Value
Whole body composition (%)	Moisture	70.9 ± 1.80	70.8 ± 0.80	68.8 ± 0.99	71.7 ± 0.57	0.1795
	Proteins	17.9 ± 0.11	18.3 ± 0.27	18.2 ± 0.6	17.8 ± 0.5	0.5180
	Lipids	7.66 ± 1.89	7.44 ± 0.55	9.82 ± 0.72	7.33 ± 0.12	0.0599
	Ash	2.78 ± 0.13	2.9 ± 0.03	3.07 ± 0.05	2.93 ± 0.2	0.1062

Nutritional indices	PER	3.24 ± 0.23	3.25 ± 0.52	3.65 ± 0.27	3.25 ± 0.53	0.5712
	GPE	58.1 ± 4.18	59.8 ± 9.57	66.6 ± 4.98	57.9 ± 9.54	0.4863
	LER	9.90 ± 0.71	9.04 ± 1.45	9.78 ± 0.73	9.12 ± 1.5	0.7322
	GLE	85.3 ± 5.47^ab^	75.4 ± 10.8^a^	109 ± 7.21^b^	78.2 ± 11.5^a^	**0.0074**

Somatic index	CF	1.11 ± 0.04	1.12 ± 0.08	1.08 ± 0.04	1.07 ± 0.05	0.1946
	VSI	3.95 ± 1.19	5.31 ± 1.75	3.78 ± 1.14	4.51 ± 0.86	0.699
	HSI	1.85 ± 0.67^ab^	2.61 ± 0.78^b^	1.74 ± 0.59^a^	2.09 ± 0.42^ab^	**0.0200**

*Note:* Data are given as the mean (*n* = 3 ± SD). In each line, different superscript letters indicate significant differences among treatments (*p* ≤ 0.05). PER = ((FBW − IBW)/protein intake). GPE = 100 × [(% final body protein × FBW) − (% initial body protein × IBW)]/total protein intake fish. LER = (FBW − IBW)/lipid intake. GLE = 100 × [(% final body lipid × FBW) − (% initial body lipid × IBW)]/total lipid intake fish. CF = 100 × (body weight, g)/(body length, cm)^3^. VSI (%) = 100 × (viscera weight/FBW). HSI (%) = 100 × (liver weight/FBW). Bold values indicate statistically significant differences between treatments (*p* < 0.05).

Abbreviations: CF, condition factor; GLE, gross lipid efficiency; GPE, gross protein efficiency; HIS, hepatosomatic index; LER, lipid efficiency rate; PER, protein efficiency ratio; VSI, viscerosomatic index.

**Table 4:** Plasma biochemistry in meagre fed with experimental diets.

Category	Parameter	C	PEA 7%	PEA 18%	PEA 27%	*p*-Value
Energy metabolism	Glucose	164 ± 33.2^b^	164 ± 28.1^b^	123 ± 36.4^ab^	104 ± 42.0^a^	**0.0013**
	Lactate	29.4 ± 11.0	28.3 ± 7.53	26.2 ± 7.01	26.7 ± 7.52	0.8413

Protein markers	Total prot	2.94 ± 0.22^a^	3.09 ± 0.15^ab^	3.28 ± 0.26^b^	3.00 ± 0.16^a^	**0.0090**
	Albumin	0.62 ± 0.05^a^	0.64 ± 0.05^ab^	0.71 ± 0.07^b^	0.68 ± 0.06^ab^	**0.0081**
	Alb/Glo ratio	0.27 ± 0.01^a^	0.26 ± 0.02^a^	0.27 ± 0.03^ab^	0.29 ± 0.03^b^	**0.0068**

Kidney function	Urea	6.74 ± 1.24	6.90 ± 1.49	7.53 ± 18	8.58 ± 1.80	0.0811
	Creatinine	0.45 ± 0.12	0.37 ± 0.08	0.33 ± 0.10	0.51 ± 0.23	0.0519
	Uric acid	0.08 ± 0.14	0.04 ± 0.03	0.05 ± 0.06	0.04 ± 0.04	0.5108

Liver function	Tot Bil	0.12 ± 0.02	0.11 ± 0.02	0.10 ± 0.04	0.10 ± 0.04	0.5819
	AST	92.0 ± 92.0	93.0 ± 61.1	104 ± 95.0	131 ± 158	0.8578
	ALT	17.0 ± 26.1	10.4 ± 44.4	12.4 ± 6.98	15.5 ± 15.8	0.8124

Enzyme activity	ALP	33.1 ± 4.86	38.3 ± 9.19	48.9 ± 30.9	53.8 ± 26.5	0.1615
	CK	1077 ± 612	1707 ± 955	2284 ± 2624	1971 ± 2310	0.5591
	LDH	445 ± 289	494 ± 322	518 ± 472	555 ± 607	0.9602

Lipid profile	Cholesterol	181 ± 21.2	201 ± 22.6	187 ± 32.7	189 ± 39.4	0.5534
	HDL	40.6 ± 5.17	42.6 ± 7.49	38.6 ± 5.41	39.9 ± 7.40	0.6203
	Triglycerides	1488 ± 234	1614 ± 434	1457 ± 611	1515 ± 755	0.9349

Minerals/ electrolytes	Ca/P ratio	16.3 ± 0.67^ab^	16.7 ± 0.46^b^	15.9 ± 0.94^a^	15.6 ± 1.11^a^	0.0467
	Ca^2+^	13.5 ± 0.74	13.9 ± 0.49	13.7 ± 0.91	12.8 ± 1.79	0.0617
	P	10.84 ± 0.89	11.4 ± 1.08	12.03 ± 1.99	11.7 ± 1.61	0.3780
	Mg	2.59 ± 0.26	2.79 ± 0.12	2.70 ± 0.27	2.59 ± 0.28	0.2841
	Fe	60.3 ± 16.2	60.6 ± 11.3	57.9 ± 20.9	56.7 ± 22.9	0.9612
	Na^+^	173 ± 4.46^b^	175 ± 3.90^b^	170 ± 2.49^ab^	167 ± 3.82^a^	**0.0010**
	K	3.04 ± 0.42	2.76 ± 0.44	3.06 ± 0.83	3.18 ± 1.16	0.6873
	Cl^−^	147 ± 2.82	148 ± 3.27	145 ± 2.12	143 ± 4.89	0.0558

*Note:* Data are given as the mean (*n* = 15/diet) ± SD. Different superscript letters indicate significant difference (One-way ANOVA *p* ≤ 0.05) between treatments. Glucose (mg/dL); lactate (mmol/L); total protein (mg/dL); albumin (g/dL); ALB/GLO (g/dL); urea (mg/dL); creatinine (mg/dL); uric acid (mg/dL); Tot Bil (mg/dL); Ast (U/L); Alt (U/L); Alp (U/L); CK (U/L); LDH (U/L); cholesterol (mg dL); HDL (mg/dL); triglycerides (mg/dL); Ca/P ratio (mg/dL); Ca^+2^ (μg/dL); P (μg/dL); Mg (μg/dL); Fe (μg dL); Na^+^ (mEq/L); K (mEq/L); Cl^–^ (mEq/L). Bold values indicate statistically significant differences between treatments (*p* < 0.05).

Abbreviations: ALB/GLO, albumin/globulin; Alp, alkaline phosphatase; Alt, alanine aminotransferase; Ast, aspartate aminotransferase; Ca^+2^, calcium; CK, creatine kinase; Cl^–^, chloride; Fe, iron; HDL, high density lipoprotein; K, potassium; LDH, lactate dehydrogenase; Mg, magnesium; Na^+^, sodium; P, phosphorus; Tot Bil, total bilirubin.

## Data Availability

The data that support the findings of this study are available from the corresponding author upon reasonable request. The data are not publicly available due to privacy and ethical restrictions.
